# Epidemiological characteristics, complications of haemodialysis patients with end-stage diabetic nephropathy in a tertiary hospital in Guizhou, China: a cross-sectional survey

**DOI:** 10.3389/fmed.2024.1418075

**Published:** 2024-10-18

**Authors:** Xingxiu Xu, Nengyuan Yang, Jingjing Da, Qian Li, Jing Yuan, Yan Zha

**Affiliations:** ^1^Renal Division, Department of Medicine, Guizhou Provincial People’s Hospital, Guizhou Provincial Institute of Nephritic and Urinary Disease, Guiyang, China; ^2^NHC Key Laboratory of Pulmonary Immunological Disease, Guizhou Provincial People’s Hospital, Guiyang, China

**Keywords:** end-stage diabetic nephropathy, vascular access, multiple complications, diabetes mellitus, diabetic nephropathy

## Abstract

**Introduction:**

In China, diabetes mellitus (DM) significantly contributes to end-stage renal disease (ESRD), necessitating treatments like hemodialysis. This study investigates hemodialysis outcomes in diabetic nephropathy patients in Guizhou Province, aiming to enhance care for this high-risk group.

**Methods:**

The cross-sectional survey design to screen haemodialysis patients with end-stage diabetic nephropathy, a structured questionnaire was designed. The collected data were collated and coded and analyzed using GraphPad Prism 9.5.0 (730).

**Results:**

Patients with end-stage diabetic nephropathy undergoing dialysis are primarily concentrated in the middle-aged and elderly population, with a higher proportion of males than females. Male patients also have a higher history of smoking and alcohol consumption compared to females. The disease progression from diabetes to diabetic nephropathy and from nephropathy to end-stage renal disease varies, with a lower dialysis vintage. Hemodialysis is the most commonly chosen treatment modality, with the highest proportion of patients selecting non-tunneled catheters. However, among patients receiving dialysis, the proportion of autogenous arteriovenous fistula (AVF) is the highest. With the increase in the duration of diabetes, the prevalence of multiple complications also increases.

**Conclusion:**

Early intervention and management strategies are crucial for diabetic nephropathy patients in Guizhou, considering the rapid disease progression to ESRD and high complication rates. The study underscores the need for targeted healthcare policies to address the unique challenges of this population, with future research required to deepen the understanding of disease mechanisms and improve patient outcomes.

## Introduction

Diabetes mellitus (DM) is one of the fastest-growing health emergencies in the world, and its prevalence in China has increased exponentially in the past 30 years (1–4). With the global rise in the prevalence of diabetes, the number of patients with diabetic nephropathy leading to End-Stage Renal Disease (ESRD) has also shown a rapid increasing trend (2). ESRD is a condition requiring lifelong treatment, typically managed through hemodialysis or kidney transplantation (3). In China, with rapid economic development and lifestyle changes, the prevalence of diabetes and its complications has significantly increased, especially in economically less developed areas such as Guizhou Province, where the issue is particularly pronounced (4). Hemodialysis is one of the most common methods for treating ESRD, but this treatment often comes with a variety of complications, such as cardiovascular diseases, infections, dialysis-related amyloidosis, etc., significantly affecting patients’ quality of life and survival rates (5). Therefore, understanding the epidemiological characteristics and complications of hemodialysis patients is crucial for improving patient management and treatment outcomes (6).

Guizhou Province, located in the southwest of China, faces unique challenges in public health and medical care services due to its special geographical location and economic conditions (7). For patients with ESRD due to diabetic nephropathy, these challenges may be even more significant. However, there are relatively few research reports on the epidemiological characteristics and complications of hemodialysis treatment for patients with end-stage diabetic nephropathy in this region. This study aims to conduct a cross-sectional survey of patients with end-stage diabetic nephropathy in a tertiary hospital in Guizhou Province, exploring the epidemiological characteristics, complications of hemodialysis treatment, and their influencing factors. We hope this research can provide a scientific basis for optimizing the treatment and management of patients with diabetic nephropathy in Guizhou Province and even in a broader region, especially in terms of improving the quality of hemodialysis treatment and reducing complications.

## Materials and methods

### Ethics statement

The study adhered to the principles of scientific morality and ethics, was approved bythe hospital ethics committee, and personal privacy and data confidentialitywere fully considered and protected. This study complied with the ethical guidelines of the1975 Declaration of Helsinki and Strengthening the Reporting of Observational Studies inEpidemiology guidelines.

### Patient population

This study adopts a cross-sectional survey design to screen haemodialysis patients with end-stage diabetic nephropathy who meet the study criteria in tertiary hospitals between October 2022 to November 2023 in Guizhou, China, as the study sample, and the participants are required to meet the diagnostic criteria of diabetic nephropathy and are receiving maintenance haemodialysis treatment. The 2022 ADA Standards of Medical Care in Diabetes and the 2022 Kidney Disease: Improving Global Outcomes (KDIGO) Clinical Practice Guidelines recommend: Diabetes mellitus (DM)D is diagnosed on the basis of elevated fasting blood glucose or 2-h blood glucose (2-h PG) values or glycated haemoglobin (A1C) levels during a 75-g oral glucose tolerance test (OGTT) (8). diabetic kidney disease (DKD) is defined as chronic kidney disease caused by diabetes mellitus, and is primarily diagnosed on the basis of a urinary albumin/creatinine ratio (UACR) above 30 mg/g and/or an estimated glomerular filtration rate (eGFR) below 60 mL-min. Diabetic kidney disease (DKD) is diagnosed by a chronic kidney disease caused by diabetes mellitus, the main diagnostic criteria being a urinary albumin/creatinine ratio (UACR) of more than 30 mg/g and/or an estimated glomerular filtration rate (eGFR) of less than 60 mL/min^−1^/(1.73 m^2^)^−1^ for a period of more than 3 months, or in some cases, in some diabetic patients. DKD patients without significant abnormalities in urinary albumin excretion, but with decreased eGFR. End-Stage Renal Disease (ESRD) are defined as DKD patients with an eGFR <30 mL/min^−1^/(1.73 m^2^)^−1^, if combined with severe complications, such as uncorrectable hypertension, intractable oedema, cardiac failure, severe anaemia, gastrointestinal toxicity, serious water electrolyte-acid–base balance disorders or an eGFR <15 mL/min^−1^/(1.73 m^2^)^−1^ (9). Inclusion Criteria: Individuals must be diagnosed with end-stage kidney disease resulting from diabetes, according to the International Classification of Diseases (ICD) standards; Participants currently receiving hemodialysis treatment or have a history of hemodialysis treatment in the past 6 months; All participants must be at least 18 years old; Participants have sufficient experience with dialysis to assess its related complications; Participants must be capable of understanding the purpose, processes, potential risks, and benefits of the study, and voluntarily agree to participate by signing the informed consent form. Exclusion Criteria: Patients currently suffering from acute kidney injury or other unstable acute medical conditions; Individuals who cannot comprehend the study information or are unable to provide informed consent due to language barriers, cognitive impairments, etc.; The treatment and prognosis for these patients may differ from those solely relying on hemodialysis; Severe cardiovascular diseases or other significant comorbidities，such as recent myocardial infarction, unstable angina, uncontrolled hypertension, severe arrhythmias, etc.; Patients currently involved in other clinical trials that could influence the outcomes of this study.

### Data collection

The study used the “Sojump” in wechat for data collection, that was collected between October 2022 to November 2023 in Guizhou, China. A structured questionnaire was designed which included basic personal information of the patients (e.g., age, gender, type of diabetes), Course of diabetes and diabetic nephropathy, comorbidities (e.g., hypertension, anaemia, cardiovascular lesions, cerebrovascular lesions, etc.) and microvascular lesions (e.g., retinopathy, peripheral neuropathy, etc.).

### Data analysis

The collected data were sorted out, coded, and analyzed by statistical methods. Data were analyzed and visualized using GraphPad Prism 9.5.0 (730) and Microsoft Office Excel 2019 to derive specific data and percentages.

## Results

### Age and gender characteristics

In this study, we evaluated the age distribution of 107 patients diagnosed with end-stage diabetic nephropathy (Table 1). The analysis revealed a distinct age-related pattern in the prevalence of the condition. Notably, a minor fraction, 6.54% (7/107), were under the age of 50. The majority of patients fell within the older age brackets: 32.71% (35/107) were aged 50–60 years, and 33.64% (36/107) belonged to the 60–70 year age group. The prevalence decreases slightly in the older cohorts, with 20.56% (22/107) aged 70–80 years and 6.54% (7/107) aged 80–90 years. Cumulatively, individuals aged 50 to 70 years constituted the largest segment of the cohort, making up 66.35% (71/107) of the population under study. The peak prevalence was observed in those aged 60–70 years, closely followed by the 50–60 year age group. In comparison, the prevalence rates were notably lower in both the youngest (<50 years) and oldest (≥80 years) age groups.

**Table 1:** The prevalence of end-stage haemodialysis patients in different age groups and pack-year smoking history and alcohol-drinking history.

Age			
**Age Group**	**Male, *n* (%)**	**Female, *n* (%)**	**Total, *n* (%)**
>40- ≤50	7 (6.54 %)	0	7 (6.54 %)
>50-≤60	28 (26.17 %)	7 (6.54 %)	35 (32.71 %)
>60-≤70	30 (28.03 %)	6 (5.61 %)	36 (33.64 %)
>70-≤80	18 (16.82 %)	4 (3.74 %)	22 (20.56 %)
>80-≤90	6 (6.54 %)	1 (0.93 %)	7 (6.54 %)
Pack-year smoking history			
**Age at smoking (in years)**		**Male, *n* (%)**	
≤10		1 (0.93 %)	
>10-≤20		11 (10.28 %)	
>20-≤30		19 (17.76 %)	
>30-≤40		15 (14.02 %)	
>40-≤50		8 (7.48 %)	
>50-≤60		2 (1.87 %)	
Alcohol-drinking history			
**Age at drink alcohol (in years)**		**Male, *n* (%)**	
≤10		1 (0.93 %)	
>10-≤20		21 (19.63 %)	
>20-≤30		13 (12.15 %)	
>30-≤40		9 (8.41 %)	
>40-≤50		4 (3.74 %)	
>50-≤60		1 (0.93 %)	

The gender distribution among 107 patients with end-stage diabetic nephropathy revealed a pronounced disparity, with males significantly outnumbering females. Specifically, males comprised 83.17% (89/107) of the cohort, whereas females represented only 16.82% (18/107). This data indicates a notably higher prevalence of end-stage diabetic nephropathy requiring haemodialysis among male patients, suggesting potential gender-related differences in the risk or progression of this condition.

### Characteristics of smoking and alcohol consumption

The smoking history among patients with end-stage diabetic nephropathy was scrutinized, revealing significant gender disparities. Specifically, none of the female patients reported a history of smoking, in stark contrast to male patients, where 47.66% reported no smoking history. Moreover, a substantial proportion of male patients, 52.33% (56/107), had a pack-year smoking history.

Historical of alcohol-drinking characteristics: According to the survey results, the rate of male patients with alcohol-drinking history was also higher than that of female patients. Specifically, the rate of female patients with non-drinker was 100%, while the rate of male patients with alcohol nondrinking was 54.21% (58/107). On the other hand, the percent age of male patients with alcohol drinking was 45.79% (49/107). This indicates that there was a relatively high percentage of alcohol consumption among the male patients whereas there was no history of alcohol consumption among the female patients.

### Type of diabetes mellitus and course of diabetes mellitus progressing to nephropathy and nephropathy progressing to end-stage renal disease

In this cohort of patients undergoing dialysis for end-stage diabetic nephropathy (Table 2), 100% (107/107) were identified as having type 2 diabetes, underscoring it as the predominant form leading to this advanced complication. Analysis of the timeline from diabetes onset to the development of nephropathy revealed a specific pattern of progression. Notably, a plurality of patients, 43.93% (47/107), developed diabetic nephropathy within a span of 1 to 5 years following their diabetes diagnosis. Comparable numbers were observed in earlier (<1 year) and intermediate (5–10 years) stages of diabetes, comprising 20.56% (22/107) and 22.43% (24/107) of the cohort, respectively. A smaller fraction progressed to nephropathy over longer durations, with 7.48% (8/107) and 4.67% (5/107) developing the condition after 10 to 15 years and over 15 years, respectively.

**Table 2:** The prevalence of progression of diabetes to diabetic nephropathy, progression of diabetic nephropathy to end-stage diabetic nephropathy, and age on dialysis.

Age of diabetes (TM)	
**Age (in years)**	***n* (%)**
≤5	4 (3.74 %)
>5-≤10	13 (12.15 %)
>10-≤15	22 (20.56 %)
>15-≤20	35 (32.71 %)
>20-≤25	17 (15.89 %)
>25-≤30	11 (10.28 %)
>30-≤35	1 (0.93 %)
>35	4 (3.74 %)
Progression of diabetes to nephropathy	
**Age (in years)**	***n* (%)**
≤1	22 (20.56 %)
>1-≤5	47 (43.93 %)
>5-≤10	24 (22.43 %)
>10-≤15	8 (7.48 %)
>15	5 (4.67 %)
Diabetic nephropathy progressing to end-stage renal disease	
**Age (in years)**	***n* (%)**
≤1	57 (53.27 %)
>1-≤5	34 (31.78 %)
>5-≤10	10 (9.35 %)
>10	6 (5.61 %)
Hemodialysis age	
**Age (in years)**	***n* (%)**
≤1	46 (42.99 %)
>1-≤5	46 (42.99 %)
>5-≤10	8 (7.48 %)
>10-≤15	7 (6.54 %)

In examining the progression from diabetic nephropathy to end-stage renal disease (ESRD) within our cohort, a distinct temporal distribution emerged. A majority, 53.27% (57/107), transitioned to ESRD within one year of nephropathy diagnosis, highlighting a rapid progression in over half of the cases. Following this, 31.78% (34/107) initiated dialysis between one to five years post-diagnosis, indicating a slower, yet significant, disease advancement. A smaller fraction, 9.35% (10/107), commenced dialysis after a period extending from five to ten years, demonstrating a prolonged disease course in a minority of patients. Lastly, 5.61% (6/107) began dialysis more than ten years following their initial nephropathy diagnosis, reflecting the longest duration of progression within the sample.

In this study, we assessed the duration of dialysis treatment among patients with diabetic nephropathy undergoing dialysis. Analysis of 107 patients revealed that a predominant 85.98% (92/107) had been on dialysis for less than five years. Notably, within this subset, an equal proportion of 42.99% (46/107) were identified in each of the sub-categories: those with less than one year of dialysis and those who had undergone dialysis for between one and five years. A smaller segment, 7.48% (8/107), had been receiving dialysis for a period ranging from five to ten years. The cohort with the longest duration of dialysis, spanning between ten and fifteen years, comprised 6.54% (7/107) of the total.

### First-time kidney replacement therapy (KRT) modality and in-dialysis modality

The survey data highlights dialysis modality preferences among patients with end-stage diabetic nephropathy. Hemodialysis emerged as the predominant initial choice, selected by 85.98% (92/107) of the cohort. Peritoneal dialysis was the second most preferred option, chosen by 12.15% (13/107) of patients. Colonic dialysis, a less common selection, was opted for by 1.87% (2/107).

Subsequent choices for ongoing dialysis modalities mirrored these preferences, with hemodialysis remaining the most prevalent among 94.39% (101/107) of patients. A combination of hemodialysis and peritoneal dialysis was selected by 5.60% (6/107), indicating a slight shift toward combined modalities for long-term management.

### First-time hemodialysis vascular access and in-dialysis vascular access selection

In our analysis of vascular access choices among patients with end-stage diabetic nephropathy undergoing their first hemodialysis session, a clear preference emerged. Non-tunneled catheter access was the predominant choice, selected by 74.77% (80/107) of the cohort. The use of a dialysis catheter with a tunnel and Dacron cuff was the second most preferred option, chosen by 13.7% (15/107) of patients. Notably, only 11.21% (12/107) opted for an autogenous arteriovenous fistula as their initial hemodialysis vascular access.

In our cohort of diabetic kidney disease (DKD) patients requiring dialysis, the preferred vascular access method for ongoing dialysis significantly favored autogenous arteriovenous fistula, chosen by 80.37% (86/107). The dialysis catheter with a tunnel and Dacron cuff was the second preference, utilized by 18.69% (20/107) of patients. Notably, half of these patients (10/20) resorted to this method following unsuccessful autogenous arteriovenous fistula surgeries and subsequent interventions such as percutaneous transluminal angioplasty (PTA) or the use of artificial vascular grafts (AVG). A solitary patient (0.93%, 1/107) selected a non-tunneled catheter as a temporary measure while awaiting the maturation of an autogenous arteriovenous fistula. This distribution underscores a strong preference for autogenous arteriovenous fistulas among DKD patients, reflecting their prioritization of long-term, sustainable vascular access for dialysis.

### Autogenous arteriovenous fistula (AVF), artificial vascular graft (AVG), percutaneous transluminal angioplasty (PTA) for vascular access

In our analysis of 107 patients undergoing dialysis for end-stage diabetic nephropathy, autogenous arteriovenous fistulas (AVF) were notably preferred for vascular access. A substantial 72.9% (78/107) of patients underwent one AVF surgery, with a success rate of 67.29% (72/107). A smaller segment, 10.28% (11/107), opted for a second AVF surgery, achieving a 7.48% success rate (8/107). Fewer still, 1.87% (2/107) and 3.74% (4/107), required three and four AVF surgeries respectively, with a cumulative success rate of 2.8% (3/107). An attempt to use an artificial vascular graft (AVG) was made by only one patient, resulting in a 100% failure rate, emphasizing the challenges associated with AVG.

Furthermore, 11.21% (12/107) of AVF users needed percutaneous transluminal angioplasty (PTA) for vascular abnormalities, with all initially successful. Subsequently, 1.87% (2/107) underwent a second, and 0.93% (1/107) a third PTA, highlighting recurrent vascular issues.

**Figure 1:**
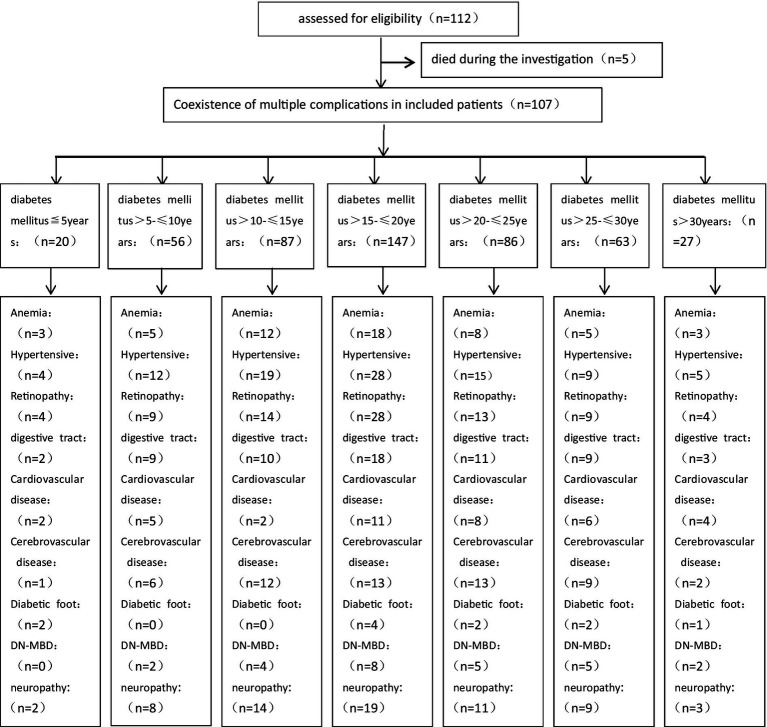
Patient flow diagram *Years of diabetes diagnosis and prevalence of complications.

**Figure 2:**
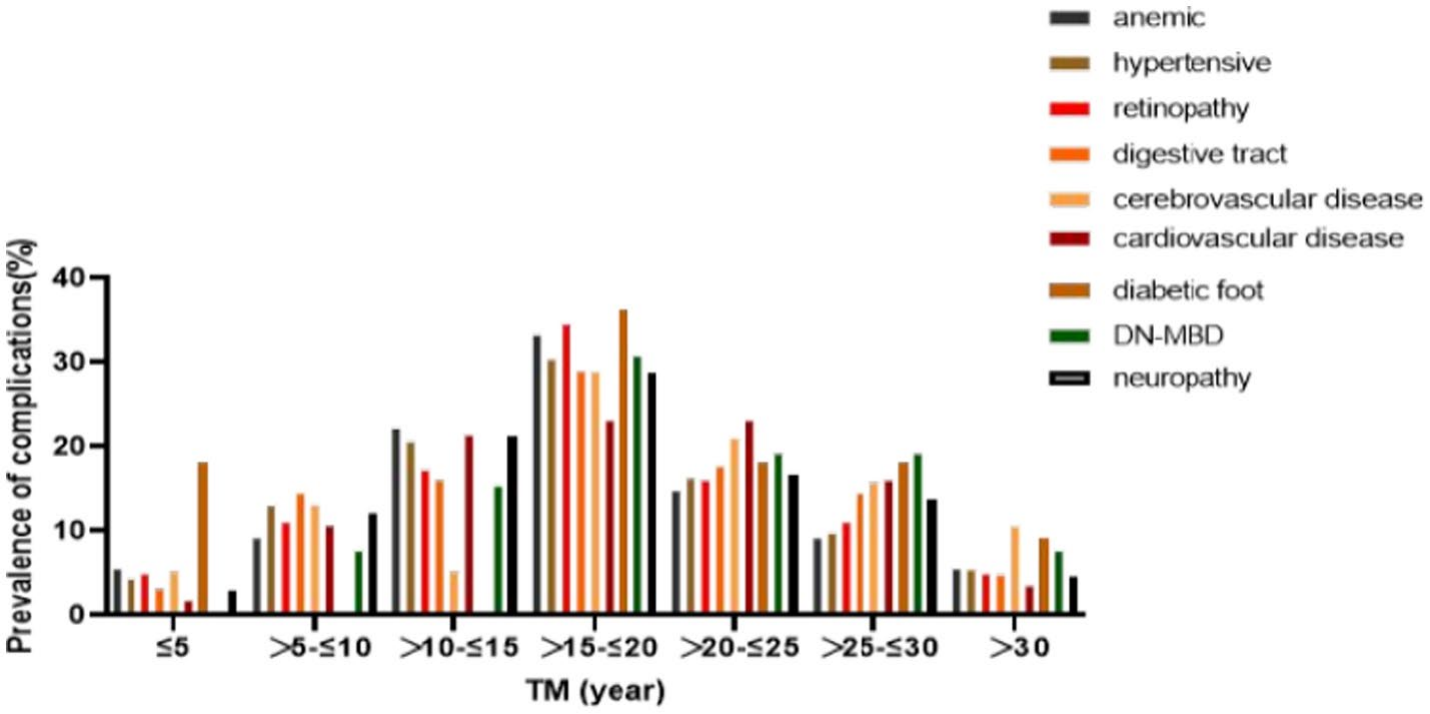
Years of diabetes diagnosis and prevalence of complications. DN-MBD: diabetic renal bone disease.

In this study, we summarized Complication rates among 107 patients diagnosed with end-stage diabetic nephropathy (Figures 1, 2) were high, with anemia (50.47%), hypertension (85.98%), retinopathy (15.7%), gastrointestinal symptoms (57.94%), cardiovascular (52.34%) and cerebrovascular diseases (52.34%), peripheral vascular disease (10.28%), diabetic bone disease (24.30%), and neuropathy (61.68%) being prevalent.

## Discussion

The past few decades have witnessed a dramatic surge in the prevalence of diabetes mellitus (DM) globally (8), with China experiencing an exponential increase, particularly highlighted in regions like Guizhou Province (9, 10). This upsurge is crucially linked to the growing incidence of end-stage renal disease (ESRD), a severe complication of DM necessitating life-sustaining treatments such as hemodialysis or kidney transplantation. The burgeoning number of ESRD cases in China, exacerbated by rapid economic development and associated lifestyle transitions, presents a significant public health challenge, especially in economically less developed areas such as Guizhou.

Our study’s findings illuminate pronounced age and gender. Disparities among the ESRD patient population undergoing hemodialysis (Table 1). Specifically, a substantial burden of the disease was observed among middle-aged and elderly patients, with those aged 50 to 70 years constituting the majority of the study cohort. This age-related pattern of prevalence points to the cumulative effects of diabetes and potentially modifiable lifestyle factors over time, emphasizing the critical window for intervention to prevent or delay the onset of diabetic nephropathy. This pattern underscores a significant burden of end-stage diabetic nephropathy predominantly among middle-aged and elderly patients, highlighting the critical need for targeted interventions in these populations.

Additionally, the stark disparity in gender distribution, with males significantly outnumbering females in the ESRD cohort, suggests underlying gender-related differences in exposure to risk factors, healthcare-seeking behavior, or biological susceptibility to diabetic complications, consistent with other studies (11–14). According to smoking and alcohol consumption Obvious difference, this stark contrast emphasizes that male patients were significantly higher than female patients, suggesting a potential risk factor for the progression of diabetic nephropathy in males (Table 1). These observations beckon a closer examination of sociodemographic influences and lifestyle determinants contributing to the observed trends, paving the way for gender-specific and age-targeted preventive strategies.

The universal manifestation of type 2 diabetes as the precursor to ESRD among the study participants underscores the global burden of this disease variant (15), reflecting broader epidemiological trends and lifestyle shifts conducive to its proliferation. The dominance of type 2 diabetes in the pathway to ESRD signals an urgent need for comprehensive diabetes management programs that encompass lifestyle modifications, regular screening for early detection of nephropathy, and integrated care approaches to manage diabetes and its complications effectively (16–18). Meanwhile, this pattern underscores the variable trajectory of diabetic nephropathy toward ESRD, with a substantial portion facing rapid deterioration, the high frequency of relatively short-term dialysis among patients with diabetic nephropathy, with a significant number initiating dialysis within 5 years of their diagnosis (Table 2), the critical window within the first decade of diabetes diagnosis for nephropathy development, indicating the need for early intervention strategies. The findings of this study, set against the backdrop of Guizhou Province’s unique challenges, highlight the importance of regionalized public health strategies that address the specific needs and risk factor profiles of the population. In doing so, it is imperative to leverage global insights on diabetes care and prevention while tailoring interventions to meet local healthcare needs and cultural contexts, thereby mitigating the burgeoning tide of diabetes and its grievous complication, ESRD.

These findings underscore hemodialysis as the primary dialysis modality (Table 3) among patients with end-stage diabetic nephropathy, reflecting its widespread acceptance and potential implications for patient care strategies and resource allocation in nephrology. In the treatment of hemodialysis, autogenous arteriovenous fistula (AVF) markedly stands out as the preferred vascular access over other forms (Table 3), including non-tunneled catheters and artificial vascular grafts (AVG) (19). This preference reflects not just a choice bias among patients and healthcare providers but also encapsulates considerations around treatment efficacy, risk of complications, and quality of life implications. The favoritism toward AVF is attributed to its lower complication rates, longer lifespan, and superior dialysis quality (20). However, the high preference for AVF has profound implications for patient outcomes, complications, and healthcare resource allocation. Initially, despite AVF’s lower risks for access-related infections and thrombosis, its creation and maturation require a significant amount of time, rendering it unsuitable for all patients, particularly those with acute dialysis needs, as a result, a significant reliance on catheter-based access for initial dialysis, with a smaller proportion of patients selecting options that are generally considered more sustainable and associated with fewer complications over the long term (Table 3). Secondly, a high AVF preference could lead to an imbalance in healthcare resource distribution, especially in resource-constrained settings like Guizhou Province, where establishing and maintaining AVFs (21) could impose additional strains on the healthcare system.

**Table 3:** First dialysis treatment modality, first haemodialysis vascular access and number of different vascular access procedures, success rate.

First KRT modality	*n* (%)	
HD	92 (85.98 %)	
PD	13 (12.15 %)	
CD	2 (1.87 %)	
Present KRT modality	*n* (%)	
HD	101 (94.39 %)	
PD+HD	6 (5.60 %)	
First hemodialysis vascular access	*n* (%)	
AVF	12 (11.21 %)	
TCC	15 (13.7 %)	
NTC	80 (74.77 %)	
Present hemodialysis vascular access	*n* (%)	
AVF	86 (80.37 %)	
TCC	20 (18.69 %)	
NTC	1 (0.93 %)	
Number of vascular accesses established and success rate		
AVF	**Times, *n* (%)**	**Success rate *n* (%)**
	1, 78 (72.9 %)	72 (67.29 %)
	2, 11 (10.28 %)	8 (7.48 %)
	3, 2 (1.87 %)	2 (1.87 %)
	4, 4 (3.74 %)	3 (2.8 %)
AVG	1, 1 (0.93 %)	0
PTA	**Times, *n* (%)**	
	1, 12 (11.21 %)	
	2, 2 (1.87 %)	
	3, 1 (0.93 %)	

KRT, kidney replacement therapy; PD, peritoneal dialysis; HD, hemodialysis; CD, colonic dialysis; NTC, non-tunneled catheter; TCC, tunnel-cuffed catheter; AVF, arteriovenous fistulae; AVG, arteriovenous graft; PTA, percutaneous transluminal angioplasty.

The challenges and success rates associated with AVF surgeries warrant thorough exploration (Table 3). While AVFs are the recommended first-line vascular access, the success of surgery is influenced by various factors, including patient vascular conditions, potential complications, and surgical techniques (22). Additionally, for patients requiring percutaneous transluminal angioplasty (PTA) to improve or maintain AVF function (23), this adds an extra layer of treatment burden, potentially affecting the quality of life and treatment efficacy. Therefore, despite the clear advantages of AVFs, a comprehensive consideration of individual patient circumstances, including their health status, urgency of dialysis, and long-term dialysis needs, is essential when selecting the vascular access.

The treatment of end-stage renal disease (ESRD) via hemodialysis, while life-sustaining, is fraught with potential complications that can significantly impact patient outcomes and quality of life. Among the study population, there were notably high rates of cardiovascular diseases, infections, and dialysis-related amyloidosis (24–28). These complications not only exacerbate the underlying disease burden but also pose significant challenges in the management and treatment of patients undergoing long-term hemodialysis (29).

What is noteworthy is that the patients with over 15 years of diabetes history showed the highest complication rates, indicating an increased risk of multiple complications with longer diabetes duration and the prevalence of having multiple complications simultaneously also increased (Figure 2). Cardiovascular diseases (30) stand as a leading cause of morbidity and mortality in patients on hemodialysis, compounded by the pre-existing cardiovascular risk associated with diabetes mellitus (31). The dialysis process itself can contribute to cardiovascular strain through fluid and electrolyte shifts, exacerbating pre-existing conditions. Infections, a prevalent risk due to vascular access sites and immunocompromised states, further complicate the clinical picture. Dialysis-related amyloidosis, caused by the accumulation of beta-2 microglobulin, underscores the long-term complications associated with hemodialysis, manifesting in joint and bone disorders that impair patient mobility and quality of life (32).

The duration of diabetes before the initiation of dialysis shows a significant correlation with the prevalence of these complications. Longer diabetes duration is associated with an increased risk of vascular and microvascular complications, which, in turn, exacerbates the challenges of managing ESRD. This relationship underscores the critical need for early intervention and comprehensive disease management strategies. By addressing diabetes and its complications early, it may be possible to delay the progression of nephropathy and reduce the burden of complications once dialysis becomes necessary.

Furthermore, gender-specific risk factors such as smoking and alcohol consumption have notable implications for the progression of diabetic nephropathy and the subsequent need for hemodialysis. The study revealed significant gender disparities in these behaviors, with male patients exhibiting a higher prevalence of smoking and alcohol consumption. These risk factors not only contribute to the development of diabetic nephropathy but also influence the spectrum and severity of complications encountered during hemodialysis treatment. Recognizing these gender-specific risk factors is essential for the development of targeted prevention and education programs. By focusing on modifiable risk factors and promoting lifestyle changes, healthcare providers can tailor their approaches to prevention and early intervention, potentially mitigating the progression of diabetic nephropathy and its complications.

The findings from this study on end-stage diabetic nephropathy patients undergoing hemodialysis in Guizhou offer significant insights that can shape both clinical practice and healthcare policy, especially in regions facing similar healthcare challenges. In clinical settings, these findings emphasize the necessity for healthcare providers in Guizhou and analogous regions to refine hemodialysis treatment protocols and complication management strategies. Recognizing the high prevalence of cardiovascular diseases, infections, and dialysis-related amyloidosis among the hemodialysis population mandates a proactive approach in monitoring, early identification, and management of these complications. Furthermore, the insights on vascular access preferences suggest a need for careful patient evaluation to choose the most suitable form of vascular access, considering individual health conditions and dialysis requirements. Clinicians should also be vigilant about gender-specific risk factors such as smoking and alcohol consumption, incorporating risk mitigation strategies into patient care plans.

The study underlines the critical role of healthcare policies tailored to the unique needs of ESRD patients in economically less developed areas. Policies should aim to address the gaps in healthcare infrastructure that impede the effective management of ESRD, such as the availability of hemodialysis units and trained nephrology specialists. Furthermore, there is a pressing need for policies that support the integration of preventive measures and early detection of diabetic nephropathy into primary healthcare services. This approach can help in mitigating the progression to ESRD and reducing the burden on dialysis services.

This study on haemodialysis patients with end-stage diabetic nephropathy, while offering valuable insights, faces limitations affecting its representativeness and generalizability. The selection of a single sample from one hospital introduces a bias, limiting the ability to generalize findings across the broader population of patients due to potential geographical and healthcare organization differences. Employing a retrospective design and relying on questionnaires for data collection further complicates this issue, introducing recall bias and compromising data accuracy and reliability. Additionally, the absence of a comparison group restricts the ability to conduct comparative analyses with other disease groups, limiting the exploration of unique differences and associated factors. Moreover, the study’s focus on only haemodialysis patients, excluding those undergoing other forms of kidney replacement therapy such as peritoneal dialysis and renal transplantation, narrows its scope. Consequently, the findings’ applicability is somewhat constrained, necessitating further studies to validate and expand upon these observations and to explore related factors more comprehensively and in depth.

## Conclusion

This study provides important insights into the hemodialysis treatment and complications among patients with end-stage diabetic nephropathy in Guizhou Province. We discovered that complications such as cardiovascular diseases, infections, and amyloidosis are prevalent and associated with the duration of diabetes and gender-specific risk factors. The research underscores the necessity for early intervention, optimization of hemodialysis treatment, and management strategies. Furthermore, tailored healthcare policies are essential for addressing the unique challenges of the region, aiming to improve patient care standards and quality of life.

## Data Availability

The original contributions presented in the study are included in the article/supplementary material, further inquiries can be directed to the corresponding author.
